# Supplementary material for: Modeling glioblastoma heterogeneity as a dynamic network of cell states
Source: Mol Syst Biol. 2021 Sep 16;17(9):e10105. doi: 10.15252/msb.202010105 (PMC8444284; doi:10.15252/msb.202010105)
Supplement: Supplementary file 6 — Source Data for Figure 5 [file MSB-17-e10105-s004.zip › Figure5A_sourcedata/GSEA_3071/hallmarks_stateII.GseaPreranked.1623742536692/HALLMARK_ESTROGEN_RESPONSE_LATE.html]

Details for gene set HALLMARK\_ESTROGEN\_RESPONSE\_LATE[GSEA]

|  || Dataset | state23071 |
| Phenotype | NoPhenotypeAvailable |
| Upregulated in class | na\_pos |
| GeneSet | HALLMARK\_ESTROGEN\_RESPONSE\_LATE |
| Enrichment Score (ES) | 0.28804877 |
| Normalized Enrichment Score (NES) | 1.5830375 |
| Nominal p-value | 0.018306635 |
| FDR q-value | 0.052804947 |
| FWER p-Value | 0.392 |
Table: GSEA Results Summary

  

Fig 1: Enrichment plot: HALLMARK\_ESTROGEN\_RESPONSE\_LATE      
 Profile of the Running ES Score & Positions of GeneSet Members on the Rank Ordered List

  

| PROBE | GENE SYMBOL | GENE\_TITLE | RANK IN GENE LIST | RANK METRIC SCORE | RUNNING ES | CORE ENRICHMENT || 1 | CDC6 |  |  | 11 | 1.631 | 0.0341 | Yes |
| 2 | GINS2 |  |  | 40 | 1.306 | 0.0567 | Yes |
| 3 | TOP2A |  |  | 72 | 1.132 | 0.0746 | Yes |
| 4 | IDH2 |  |  | 78 | 1.117 | 0.0986 | Yes |
| 5 | RBBP8 |  |  | 86 | 1.100 | 0.1217 | Yes |
| 6 | STIL |  |  | 108 | 1.031 | 0.1398 | Yes |
| 7 | CAV1 |  |  | 134 | 0.988 | 0.1559 | Yes |
| 8 | PLK4 |  |  | 205 | 0.870 | 0.1582 | Yes |
| 9 | TFF3 |  |  | 225 | 0.851 | 0.1728 | Yes |
| 10 | HPRT1 |  |  | 351 | 0.709 | 0.1578 | Yes |
| 11 | FKBP5 |  |  | 375 | 0.695 | 0.1678 | Yes |
| 12 | IGFBP4 |  |  | 401 | 0.678 | 0.1769 | Yes |
| 13 | NXT1 |  |  | 438 | 0.657 | 0.1828 | Yes |
| 14 | FLNB |  |  | 488 | 0.633 | 0.1850 | Yes |
| 15 | PERP |  |  | 496 | 0.630 | 0.1975 | Yes |
| 16 | SLC9A3R1 |  |  | 497 | 0.630 | 0.2117 | Yes |
| 17 | PAPSS2 |  |  | 529 | 0.612 | 0.2179 | Yes |
| 18 | BTG3 |  |  | 540 | 0.607 | 0.2291 | Yes |
| 19 | MEST |  |  | 563 | 0.593 | 0.2370 | Yes |
| 20 | PPIF |  |  | 639 | 0.560 | 0.2311 | Yes |
| 21 | MICB |  |  | 655 | 0.550 | 0.2398 | Yes |
| 22 | PRSS23 |  |  | 666 | 0.546 | 0.2497 | Yes |
| 23 | XRCC3 |  |  | 669 | 0.545 | 0.2615 | Yes |
| 24 | GAL |  |  | 708 | 0.530 | 0.2640 | Yes |
| 25 | MYOF |  |  | 730 | 0.523 | 0.2707 | Yes |
| 26 | SLC29A1 |  |  | 789 | 0.502 | 0.2676 | Yes |
| 27 | FKBP4 |  |  | 806 | 0.495 | 0.2748 | Yes |
| 28 | BCL2 |  |  | 813 | 0.494 | 0.2845 | Yes |
| 29 | CDC20 |  |  | 844 | 0.486 | 0.2880 | Yes |
| 30 | MOCS2 |  |  | 1244 | 0.387 | 0.1978 | No |
| 31 | FOXC1 |  |  | 1271 | 0.383 | 0.2001 | No |
| 32 | RNASEH2A |  |  | 1341 | 0.369 | 0.1913 | No |
| 33 | SLC7A5 |  |  | 1434 | 0.351 | 0.1764 | No |
| 34 | GALE |  |  | 1476 | 0.344 | 0.1740 | No |
| 35 | LSR |  |  | 1656 | 0.316 | 0.1367 | No |
| 36 | ATP2B4 |  |  | 1713 | 0.308 | 0.1298 | No |
| 37 | CCND1 |  |  | 1753 | 0.304 | 0.1270 | No |
| 38 | UGDH |  |  | 1797 | 0.299 | 0.1231 | No |
| 39 | SGK1 |  |  | 1806 | 0.298 | 0.1279 | No |
| 40 | TPBG |  |  | 1914 | 0.284 | 0.1077 | No |
| 41 | XBP1 |  |  | 1995 | 0.273 | 0.0941 | No |
| 42 | SORD |  |  | 2077 | 0.265 | 0.0800 | No |
| 43 | HSPA4L |  |  | 2203 | 0.252 | 0.0546 | No |
| 44 | CXCL12 |  |  | 2350 | -0.264 | 0.0244 | No |
| 45 | PRKAR2B |  |  | 2550 | -0.293 | -0.0183 | No |
| 46 | TOB1 |  |  | 2580 | -0.298 | -0.0188 | No |
| 47 | TSPAN13 |  |  | 2706 | -0.318 | -0.0426 | No |
| 48 | CPE |  |  | 2994 | -0.371 | -0.1054 | No |
| 49 | FARP1 |  |  | 3001 | -0.373 | -0.0985 | No |
| 50 | SLC26A2 |  |  | 3212 | -0.431 | -0.1408 | No |
| 51 | RPS6KA2 |  |  | 3264 | -0.444 | -0.1434 | No |
| 52 | FABP5 |  |  | 3513 | -0.549 | -0.1925 | No |
| 53 | OLFM1 |  |  | 3521 | -0.554 | -0.1817 | No |
| 54 | CELSR2 |  |  | 3550 | -0.570 | -0.1758 | No |
| 55 | CD9 |  |  | 3567 | -0.581 | -0.1666 | No |
| 56 | ALDH3A2 |  |  | 3609 | -0.609 | -0.1630 | No |
| 57 | HOMER2 |  |  | 3625 | -0.622 | -0.1527 | No |
| 58 | ID2 |  |  | 3664 | -0.654 | -0.1473 | No |
| 59 | MDK |  |  | 3706 | -0.691 | -0.1419 | No |
| 60 | RAB31 |  |  | 3757 | -0.737 | -0.1376 | No |
| 61 | COX6C |  |  | 3792 | -0.782 | -0.1284 | No |
| 62 | FOS |  |  | 3911 | -0.966 | -0.1358 | No |
| 63 | CHPT1 |  |  | 3923 | -1.006 | -0.1158 | No |
| 64 | NBL1 |  |  | 3992 | -1.204 | -0.1055 | No |
| 65 | ASCL1 |  |  | 4061 | -1.762 | -0.0825 | No |
| 66 | CXCL14 |  |  | 4066 | -1.885 | -0.0409 | No |
| 67 | CKB |  |  | 4075 | -2.149 | 0.0057 | No |
Table: GSEA details [plain text format]

  

Fig 2: HALLMARK\_ESTROGEN\_RESPONSE\_LATE: Random ES distribution      
 Gene set null distribution of ES for **HALLMARK\_ESTROGEN\_RESPONSE\_LATE**
